# Acute Myeloid Leukemia Harboring the t(16;21)(p11;q22) Translocation Treated With Venetoclax Plus Azacitidine After Cord Blood Transplantation

**DOI:** 10.7759/cureus.42215

**Published:** 2023-07-20

**Authors:** Kazuaki Teshima, Sho Ikeda, Ko Abe, Masahiro Yamada, Naoto Takahashi

**Affiliations:** 1 Hematology, Hiraka General Hospital, Yokote, JPN; 2 Hematology, Nephrology, and Rheumatology, Akita University Graduate School of Medicine, Akita, JPN

**Keywords:** translocation, azacitidine, venetoclax, hematopoietic stem cell transplantation, acute myeloid leukemia

## Abstract

A 62-year-old female was diagnosed with acute myeloid leukemia (AML) with t(16;21)(p11;q22). She achieved complete hematological remission after induction therapy and underwent umbilical cord blood stem cell transplantation (CBT). At 150 days after the CBT, a bone marrow examination revealed relapse. We treated the patient with venetoclax plus azacitidine as salvage therapy. After five cycles of venetoclax and azacitidine therapy, the patient died due to disease progression. The prognosis of AML with t(16;21)(p11;q22) is very poor owing to the high rate of early relapse even after hematopoietic stem cell transplantation. Therefore, a novel therapeutic approach is required to improve patient outcomes.

## Introduction

The chromosomal translocation of t(16;21)(p11;q22) shows an incidence of 1% in acute myeloid leukemia (AML) [1]. AML with t(16;21)(p11;q22) has been reported to be associated with poor prognosis even in patients undergoing hematopoietic stem cell transplantation (HSCT) [2,3]. The t(16;21)(p11;q22) is very rare; thus far, no studies have reported venetoclax (VEN) treatment for AML patients with t(16;21)(p11;q22). Here, we report a patient of AML with t(16;21)(p11;q22) who experienced early relapse after the first complete hematological remission after umbilical cord blood stem cell transplantation (CBT) and received VEN plus azacitidine (AZA) as salvage therapy. The purpose of this study is to share our experience in treating AML with VEN and AZA, highlighting our therapeutic approach to AML with t(16;21)(p11;q22).

## Case presentation

**Figure 1:**
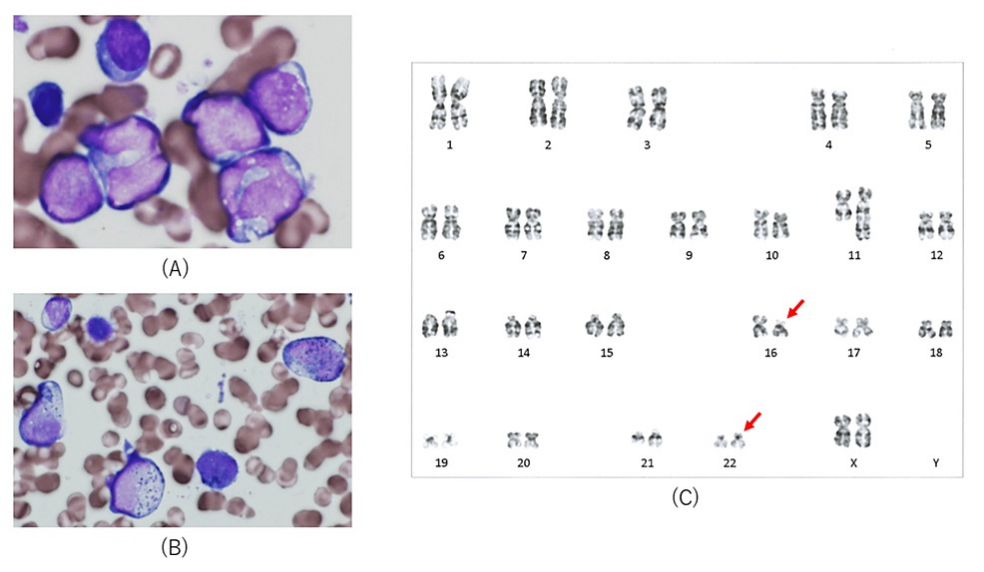
Bone marrow aspiration at diagnosis. The images show (A) bone marrow aspirate showing blasts (May-Giemsa staining ×1,000), (B) blasts were myeloperoxidase-positive (myeloperoxidase staining ×200), and (C) chromosomal banding of bone marrow cells with t(16;21)(p11;q22) (arrows).

A 62-year-old female presenting with fever was admitted to our hospital. Peripheral blood evaluation showed the following: white blood cell count of 10.1×10^3^/µL (reference range: 3.3×10^3^ to 8.6×10^3^/µL), blast cell count 84%, hemoglobin level of 11.2 g/dL (reference range: 11.6-14.8 g/dL), and platelet count of 85×10^3^/µL (reference range: 158×10^3 ^to 348×10^3^/µL). Bone marrow aspiration confirmed the diagnosis of AML (Figures 1A, 1B). Flow cytometry analyses revealed CD56, CD13, and CD33 positivity in leukemic cells. Chromosomal banding of the bone marrow cells revealed t(16;21)(p11;q22) (Figure 1C).

**Figure 2:**
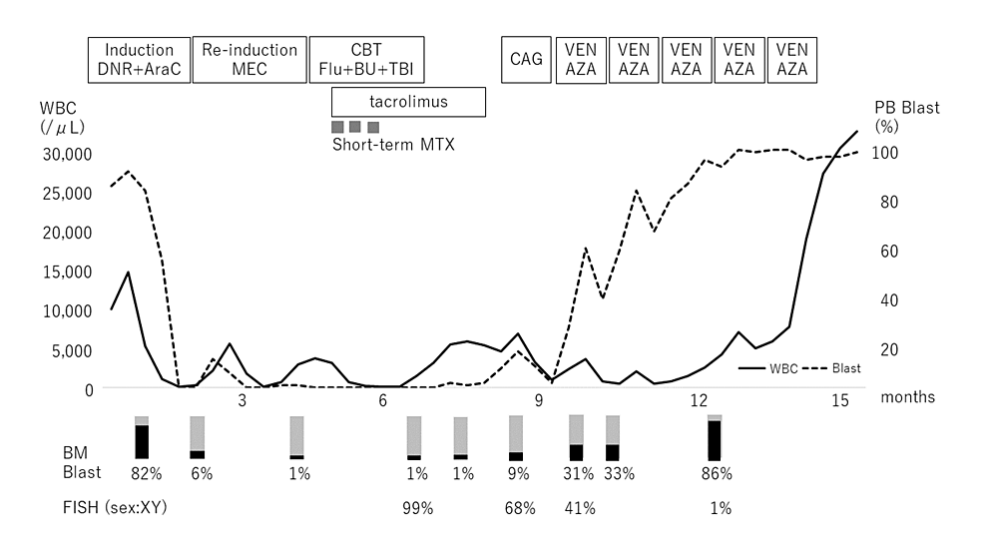
Clinical course of our case. DNR: daunorubicin; AraC: cytarabine; MEC: mitoxantrone, etoposide, cytarabine; CBT: cord blood stem cell transplantation; Flu: fludarabine; BU: busulfan; TBI: total body irradiation; CAG: cytarabine, aclarubicin, granulocyte-colony stimulating factor; VEN: venetoclax; AZA: azacitidine; MTX: methotrexate; WBC: white blood cell; PB: peripheral blood; FISH: fluorescence in situ hybridization

The clinical course of our case was shown in Figure 2. The patient was administered cytarabine and daunorubicin in the form of induction chemotherapy and failed to achieve complete remission. Therefore, the patient received re-induction therapy with mitoxantrone, etoposide, and cytarabine and achieved complete hematological remission. Subsequently, umbilical CBT was performed after the first complete hematological remission (three-allele mismatch, male, CD34-positive cell count: 1.94×10^6^ cells/kg). The conditioning regimen was composed of fludarabine (30 mg/m^2^ daily for six days), busulfan (4 mg/kg daily for two days), and total body irradiation (4 Gy). Graft-versus-host disease prophylaxis was administered with tacrolimus and short-term methotrexate. Fluorescence in situ hybridization of sex chromosomes showed complete chimerism on day 93. Moreover, t(16;21)(p11;q22) was undetectable in G-banding bone marrow cells. At 150 days after CBT, bone marrow examination revealed hematological relapse owing to the proliferation of t(16;21)(p11;q22)-harboring blast cells. There is no donor candidate for second transplantation. And we had no choice but to treat with gilteritinib or quizartinib as FLT3-ITD mutation was not detected. Therefore, a combination of VEN and AZA was administered as salvage therapy after low-dose cytarabine and aclarubicin plus a granulocyte colony-stimulating factor therapy. However, although five cycles of the VEN and AZA combination were administered, a hematological response was not observed. The patient died due to disease progression 15 months after the initial diagnosis.

## Discussion

Our case highlights AML harboring the t(16;21)(p11;q22), which was shown to be 0.94% in 1,277 AML patients [1]. The t(16;21)(p11;q22) translocation has been shown to be associated with poor outcomes [2,3]. A previous report indicated that AML with t(16;21)(p11;q22) had a high relapse rate (75%) and mortality rate (75%) [1]. Another report showed that the median overall survival period was 16 months (6-38 months) among 19 patients [4]. AML with t(16;21)(p11;q22) often expresses CD56, and expression of the CD56 has been reported to be associated with an unfavorable prognosis [5].

Therefore, performing HSCT should be considered in eligible AML patients with t(16;21)(p11;q22) who are in complete remission. However, the outcomes of HSCT among AML patients with t(16;21)(p11;q22) have been mentioned to be poor [3]. Despite undergoing HSCT during remission, 12 of the 14 studied patients eventually died [3]. Thus, HSCT alone is not a fully effective therapy for AML with t(16;21)(p11;q22), and an alternative therapeutic approach is required to improve patient outcomes.

Several reports have shown that epigenetic therapies, such as AZA, can be used as maintenance therapy after HSCT for AML with t(16;21)(p11;q22) [6,7]. After HSCT, AZA administration can stimulate a CD8-positive T-cell signal against tumor cells, and increase the effect of a graft-versus-leukemia (GVL) response [8]. Another report showed that administration of AZA could delay hematological relapse in AML patients who are at a high risk of relapse [9]. Therefore, AZA administration as maintenance therapy after HSCT for AML with t(16;21)(p11;q22) can be useful in clinical settings to achieve epigenetic and GVL effects [10]. In addition to AZA, several reports have shown that VEN is effective in relapsed and refractory AML [11]. Therefore, we administered a combination of VEN and AZA to induce a GVL response after HSCT at the time of relapse. However, the efficacy of VEN and AZA combination therapy was limited given the gradual decrease in donor chimerism over the clinical course. After five cycles of VEN and AZA, the patient died due to disease progression.

The combination of VEN and AZA followed by HSCT was reported to be effective in newly-diagnosed AML patients [12]. This could indicate that the combination of VEN and AZA should be administered at an early stage rather than at the time of relapse. Therefore, in the case of AML with t(16;21)(p11;q22), which is associated with very poor prognosis, we recommend administering the VEN and AZA combination as maintenance therapy immediately after HSCT following the first complete remission.

## Conclusions

The t(16;21)(p11;q22) in AML cases is associated with poor prognosis. Therefore, it is necessary to establish an alternative therapeutic approach to improve patient outcomes. A combination of VEN and AZA could be administered as maintenance therapy immediately after HSCT following the first complete remission. Evidence related to AML with t(16;21)(p11;q22) should be accumulated to facilitate the development of new treatment strategies.
